# Evidence of Altered Functional Connectivity at Rest in the Writing Network of Children with Dyslexia

**DOI:** 10.3390/brainsci12020243

**Published:** 2022-02-10

**Authors:** Claire Gosse, Laurence Dricot, Marie Van Reybroeck

**Affiliations:** 1 Psychological Sciences Research Institute, UCLouvain, 1348 Louvain-la-Neuve, Belgium; marie.vanreybroeck@uclouvain.be; 2 Institute of NeuroScience, UCLouvain, 1200 Brussels, Belgium; laurence.dricot@uclouvain.be

**Keywords:** developmental dyslexia, graphomotor difficulties, writing, fMRI, resting-state functional connectivity, graphemic/motor frontal area

## Abstract

Aim. Handwriting abilities in children with dyslexia (DYS) are not well documented in the current literature, and the presence of graphomotor impairment in addition to spelling impairment in dyslexia is controversial. Using resting-state functional connectivity (RSFC), the present study aims to answer the following question: are there markers of graphomotor impairment at rest in DYS children? Method. The participants were children with DYS and typically developing (TD) children (*n* = 32) from French-speaking primary schools (*M_age_* = 9.3 years). The behavioural evaluation consisted of spelling and handwriting measures. Participants underwent a resting-state fMRI scan. Results. Analyses of RSFC focused on a brain region responsible for graphomotor processes—the graphemic/motor frontal area (GMFA). The RSFC between the GMFA and all other voxels of the brain was measured. Whole-brain ANOVAs were run to compare RSFC in DYS and TD children. The results demonstrated reduced RSFC in DYS compared to TD between the GMFA and brain areas involved in both spelling processes and motor-related processes. Conclusions. For the first time, this study highlighted a disruption of the writing network in DYS. By identifying functional markers of both spelling and handwriting deficits at rest in young DYS participants, this study supports the presence of graphomotor impairment in dyslexia.

## 1. Introduction

Developmental dyslexia is a neurobiological disorder characterised by deviant literacy development, [1,2,3,4,5] affecting approximately 10% of the French-speaking population, the language of the present study. The symptoms of dyslexia occur despite normal intelligence, adequate education and the absence of sensory deficits, and they persist across a lifespan. In addition to reading impairment, DYS children have a severe and persistent impairment in spelling, which remains the most visible symptom of dyslexia in adults [6,7]. Past research has repeatedly demonstrated that DYS children struggle with all writing activities that are carried out every day in school, such as copying tasks [8,9], dictation tasks [10] and text composition [11]. Beyond school, writing difficulties can negatively impact children’s development in all aspects as poor writing abilities can have far-reaching consequences for children’s self-esteem and academic achievement [12,13]. Despite the importance of mastering writing and the severity and persistence of DYS-related writing impairment, current research on dyslexia focuses substantially more on reading difficulties than on writing difficulties. In particular, a gap in the literature concerns the presence of graphomotor difficulties in children with dyslexia in addition to their spelling impairment.

Indeed, handwriting difficulties have been repeatedly observed in DYS samples using measures of handwriting speed and/or handwriting legibility [9,14,15,16,17]. However, their origin is not yet understood as poor handwriting performance may be a consequence of spelling impairment [11,18] or may instead indicate a graphomotor deficit [10,14]. Recently, abnormal brain activation of graphomotor regions in DYS children has been demonstrated during an fMRI handwriting task [19]. These results strengthened the need to focus on writing difficulties in dyslexia by considering not only spelling but also graphomotor processes. To date, this task-fMRI study constitutes the only investigation of graphomotor abilities in dyslexia using MRI data. The use of complementary neuroimaging techniques would allow these conclusions to be confirmed. Resting-state functional connectivity (RSFC) is another type of fMRI sequence that provides information about the functional organisation of brain networks at rest [20,21]. As it does not require participants to perform any task inside the MRI, RSFC has considerable methodological advantages [21,22,23]. Nevertheless, RSFC currently appears to be underutilised in the field of research in dyslexia. To date, the few studies that have investigated RSFC in dyslexic samples have successfully identified functional markers of reading impairment at rest [23,24,25]. However, no study has focused on measuring RSFC in the writing network of individuals with dyslexia. By using RSFC data in DYS and typically developing (TD) children, the present study aimed to answer the following research question: are there functional markers of graphomotor dysfunction visible at rest in DYS children? By measuring functional connectivity with a brain region responsible for handwriting, i.e., the graphemic/motor frontal area (GMFA) [26,27], in children with and without dyslexia, this study has the potential to significantly improve our understanding of writing impairment in dyslexia.

### 1.1. Writing Difficulties in Dyslexia

Due to a phonological deficit, DYS children struggle with developing literacy from the very start of reading and writing instruction [2,5,28]. The most described and studied behavioural manifestations of dyslexia are less accurate, slower and hesitant reading. However, in addition to their reading impairment, children with dyslexia face severe and persistent spelling difficulties [5,6,7]. At the beginning of primary school, DYS children have difficulty mastering the correspondences between phonemes and graphemes [29]. Spelling difficulties in dyslexia are persistent and considered the most durable visible symptom of dyslexia throughout life [7]. More concretely, misspellings in individuals with dyslexia consist of nonphonetic errors such as inversion, substitution or omission of letters [30]. DYS children also produce conventional phonetic misspellings, referred to as orthographic errors (e.g., *readable* spelt *readible*), and more variations of spellings for the same word (e.g., *college* spelt as both *collage* and *colege* by the same writer) [31,32].

In addition to spelling accuracy, an important aspect of writing development concerns the mastery of handwriting [33,34], which is a complex ability relying on graphomotor processes [33,35,36,37,38]. As featured in the psychomotor theory of writing by Van Galen [34] (see [39] for a more recent elaboration), the graphomotor processes are divided into distinct modules: selection of the sensorimotor programmes associated with each letter stored in memory; adjustment of the size parameter for each allograph; and recruitment of the arm and hand muscles to trace the letters.

In past literature, research has highlighted the presence of graphomotor difficulties in dyslexia as characterised by slowness and poor legibility [9,10,14,15,16]. Understanding DYS children’s graphomotor difficulties has been the subject of a growing number of studies in recent years, e.g., [10,14,18,19]. However, knowing that handwriting involves a multitude of processes and a close relationship with spelling, disentangling the origin of these handwriting difficulties is challenging. Consequently, several hypotheses have been proposed to explain these handwriting difficulties in DYS children.

On the one hand, authors have supported the idea that DYS children show poorer handwriting abilities as a direct consequence of spelling impairment. Indeed, results have demonstrated that DYS children execute handwriting at the same speed as their peers but pause more during writing tasks because of their spelling impairment [8,11]. Such observations led authors to conclude that DYS children are slow at completing writing tasks because of the spelling deficit, not supporting the idea that they have actual graphomotor difficulties [11,18,40]. Moreover, as highlighted by a recent study, DYS children’s poor handwriting performance may be a consequence of their reduced writing practice because of their literacy impairments [18]. Indeed, because spelling is difficult for DYS children, they may write on fewer occasions than their peers.

On the other hand, recent findings have defended the idea that DYS-related deficits in writing concern both spelling and graphomotor processes. Indeed, the results have revealed handwriting difficulties in DYS children compared to TD children that could not be attributed to children’s spelling difficulties as they occurred during a task that did not require orthographic skills, i.e., the name and surname writing task [14]. Moreover, another study demonstrated that DYS children were more influenced by the graphic structure of words, leading to handwriting difficulties when writing words that contain graphically complex segments [10]. More precisely, DYS children in grades 3 and 4 made more graphical errors, such as letter distortions, than younger TD children that were matched for spelling ability.

To conclude, several studies have highlighted the presence of handwriting difficulties in DYS children compared to their TD peers [9,10,11,14,19]. To date, the origin of graphomotor difficulties in dyslexia is still debated. Indeed, because writing is a complex ability involving spelling and graphomotor processes [41], several plausible causes may lead to handwriting difficulties. Therefore, more research is needed to understand the origin of handwriting difficulties in dyslexia.

### 1.2. The Contribution of fMRI Techniques to Investigate Dyslexia

Overall, past fMRI studies have identified regions in which DYS has shown abnormal strength of functional activation in both children and adults [42,43,44,45]. Such experiments required participants, i.e., typical readers compared to DYS individuals, to perform various literacy tasks inside the MRI. However, even if task-fMRI studies are undeniably valuable and informative for the field of literacy development and disability, they present several limitations. First, while literacy abilities require a multitude of tasks to be comprehensively assessed, neuroimaging studies have necessarily focused on one subskill during fMRI (e.g., word and pseudoword matching in [46]; writing under dictation in [19]). Moreover, knowing that participants with dyslexia presumably dislike performing literacy tasks because of their disability, performance during the fMRI task and the functional data acquired can be biased by negative emotional factors.

With these issues in mind, RSFC appears to be a complementary fMRI technique that can overcome these methodological challenges. RSFC can be defined as a significantly correlated signal between functionally related regions in the absence of any stimulus, providing an evaluation of functional brain organisation [47]. RSFC is based on the principle that functionally related regions, e.g., regions forming literacy networks, are known to show greater connectivity with each other at rest [21]. A small number of studies have used RSFC to investigate the functional organisation of the reading network in DYS compared to TD individuals. Overall, these studies revealed reduced RSFC in regions of the reading network in DYS participants (i.e., between the left intraparietal sulcus and the left middle frontal gyrus [25]; between the left posterior temporal areas and the inferior frontal gyrus [23]; and between the left and the right inferior frontal gyri [24]). These findings provided evidence of the presence of functional markers of dyslexia at rest underlying the observed reading deficit. To date, no neuroimaging study has addressed the issue of writing difficulties in DYS children using RSFC in the writing network.

The adult brain writing network has been identified and presented in two meta-analyses [27,37]. The writing network is composed of regions of the left hemisphere responsible for either the spelling processes (i.e., the fusiform gyrus, inferior temporal gyrus, inferior frontal gyrus, superior temporal sulcus, supramarginal and superior temporal gyri) or the graphomotor processes (i.e., the parietal cortex, anterior and posterior regions of the right cerebellum, the graphemic/motor frontal area (GMFA), also known as the Exner area [26]). The cerebellar graphomotor areas play a role in the motor control of complex finger movements. However, their specificity for handwriting production has not been fully demonstrated as their recruitment was highlighted during other motor-related activities [27,37]. Moreover, an increasing number of studies focusing on the cerebellum have revealed that this is a multifunctional structure that connects with most parts of the brain [48,49,50]. Conversely, the GMFA’s role has been demonstrated to be specific to graphomotor production. The left GMFA seed region is located in the left premotor cortex (BA6) in the posterior part of the middle frontal gyrus. Authors have revealed that a lesion of the GMFA negatively impacted the formation of letters [51,52,53]. Further research has led to refining our understanding of the GMFA, which is in charge of translating orthographic information into graphomotor plans [27,37,54]. Finally, motor regions (i.e., primary motor area and supplementary motor area, for example) are also recruited for graphomotor production, but they are not specific to the act of writing [27,37].

This network was recently investigated for the first time in TD children using task-fMRI [55], revealing that the regions recruited by children during writing (aged from 8 to 11 years old) corresponded to the adult writing network [27,37]. Of particular interest to the current study, the network was also investigated in children with and without dyslexia for the first time during an fMRI dictation task [19]. The results highlighted functional differences in the writing network [27,37] between DYS and TD children in regions not only responsible for spelling but also for graphomotor and motor-related processes. These dysfunctions were characterised by less brain activation in DYS children than in TD children, particularly in cerebellar regions that play a role in controlling complex finger movements during handwriting. To date, this task-fMRI experiment constitutes the only investigation of DYS children’s handwriting abilities using neuroimaging data. Graphomotor dysfunction in DYS children needs to be confirmed and further defined by robust complementary evidence. Importantly, no study has ever investigated the writing network at rest in children with dyslexia. The use of RSFC constitutes an innovative approach for understanding graphomotor difficulties in children with DYS.

### 1.3. The Present Study

To date, writing difficulties in dyslexia are not as well documented as reading deficits. An important gap in the current literature concerns the issue of graphomotor impairment in dyslexia. Indeed, while the presence of severe spelling impairment in dyslexia is undeniable, the frequently observed handwriting difficulties in DYS children are not well understood [10,11,14,15,18]. As writing ability involves a multitude of cognitive and motor processes that influence each other [36,38,56,57,58], disentangling the issue of the origin of handwriting difficulties is challenging. The use of neuroimaging techniques to examine the writing brain network [27,37] in DYS children constitutes a promising avenue to better understand their writing difficulties. Remarkably, while reduced RSFC has been highlighted in DYS samples in the reading network [23,24,25], no study has ever investigated RSFC in the brain network responsible for writing in DYS children.

The aim of the present study was to test the hypothesis of graphomotor impairment in dyslexia by comparing RSFC in DYS and TD children. Sixteen DYS children participated in this study. They were matched on chronological age with 16 TD children. All children underwent a behavioural evaluation, including measures of spelling accuracy and handwriting, and a neuroimaging evaluation, including a resting-state fMRI sequence. Due to the key role of the GMFA region in graphomotor processes (i.e., the region responsible for transforming orthographic information into graphic traces, [26,27,37]), we measured RSFC between this area and the rest of the brain in all participants. We used RSFC to locate the brain areas with which the GMFA connects differently in DYS children compared to TD children.

Precisely, this study will address the following research question: are there functional markers of graphomotor impairment visible at rest in DYS children?

We expect our analyses to highlight differences between DYS and TD children in functional coupling of the GMFA. Differences in RSFC between the GMFA and other brain regions are interpreted in relation to their potential involvement in spelling, reading and graphomotor processes [27].

We hypothesise that DYS children will show decreased functional connectivity patterns with the GMFA seed region compared to TD children during rest. Indeed, similar to how a reduction in RSFC in the reading network underlies DYS-related reading impairment [23,24,25], we expect RSFC in writing regions to be in the sense of a reduction in the DYS group compared to the TD group. More precisely, knowing that DYS children have a severe spelling deficit, we expect to highlight reduced RSFC between the GMFA and spelling-related regions in DYS. In addition, revealing a reduction in RSFC between the GMFA and motor-related areas would support the hypothesis of the presence of additional graphomotor difficulties in DYS.

This RSFC approach has the potential to identify markers of writing impairment at rest in DYS children, which will contribute to filling the gap in the current literature regarding the origin of DYS children’s poor handwriting performance [10,11,14,15,18]. More broadly, RSFC data of the writing network may contribute to improving our current knowledge of the brain basis of dyslexia in a young population.

## 2. Method

### 2.1. Participants

Participant recruitment targeted DYS children and TD children. This took place in six ordinary primary schools in the French-speaking region of Belgium. All children and parents provided informed consent for inclusion before they participated in the study. The experiment was carried out with respect to the ethical standards of the Declaration of Helsinki and received approval by the Ethics Committee of the University Hospital of Saint-Luc (number: B403201942022). Thirty-two children participated in the present experiment according to a series of inclusion criteria (native French-speaking, aged between 7 and 10 years old and right-handed) and exclusion criteria (sensory deficit, a history of brain damage, medication use, learning disability other than dyslexia and metal in the body). Half of the sample was composed of DYS children (*n* = 16; *M_age_* = 9.38 years, *SD* = 1.24, *Range* = 7.14–11.25) who had all been diagnosed by a speech and language therapist. Children in the TD group had no history of learning difficulty (*n* = 16; *M* = 9.32 years, *SD* = 0.96, *Range* = 7.88–10.85). Children in both groups presented grade-adequate standardised scores in nonverbal IQ (DYS: *M* = 0.21, *SD* = 0.75, *Range* = −1.00–1.33; TD: *M* = 0.46, *SD* = 0.74, *Range* = −1.33–2.00) and receptive vocabulary (DYS: *M* = 1.18, *SD* = 0.66, *Range* = 0.07–2.20; TD: *M* = 1.22, *SD* = 0.88, *Range* = −0.13–2.87), as measured by the Matrix Reasoning Subtest of the WISC-IV [59] and the French adaptation of the Peabody Pictures Vocabulary Test [60], respectively. Group comparisons ensured that DYS and TD children were equivalent in age (*t(*30) = 0.17, *p =* 0.870), nonverbal IQ (*t(*30) = −0.96, *p =* 0.346) and receptive vocabulary (*t*(30) = −0.14, *p =* 0.890). Group comparisons performed on children’s behavioural performances (see 3.3 for details on the tasks used) revealed that TD children outperformed DYS children in all reading and spelling measures and in handwriting legibility (all *p*s < 0.05). The performance of the children in the behavioural evaluation is presented in Table 1.

### 2.2. Procedure

The current experiment was composed of two phases: a behavioural evaluation and MRI scanning. Children performed both phases of the evaluation in a narrow time interval in February 2020. Cognitive assessment tasks were individually administered to each participant either at home or at school. The MRI scan sessions took place at the university hospital on weekends. Anatomic, functional (task and resting-state) and multishell diffusion sequences were acquired at Cliniques Universitaires Saint-Luc (UCLouvain, Brussels, Belgium). For the purpose of this paper, we exclusively focused on RSFC data. The results concerning structural and task-fMRI data have been published in a previous paper [19]. Scanning started with acquiring a T1-weighted image, followed by resting-state fMRI, during which participants were scanned with their eyes closed and were instructed to allow their thoughts to freely roam and to think of nothing in particular. Due to the young age of the participants, we used a playful protocol for the whole MRI scan session inspired by a previous study conducted on young children with dyslexia [61]. The rocket protocol consisted of immersing the children in a space mission theme. The MRI scanner was presented to children as a type of rocket ship, and the children and the experimenters were wearing an astronaut suit during the pre-scanning preparation and the MRI data acquisition (for a complete description of the rocket protocol, see [19]).

### 2.3. Behavioural Evaluation

#### 2.3.1. Word Reading (Accuracy and Speed)

Reading ability was assessed with a standardised test from the Batterie Analytique du Langage Ecrit (Analytic Battery of Written Language) [62]. For the reading assessment, children read several items aloud: 20 irregular words, 20 regular words and 20 nonwords. The children had to read the items aloud as quickly as possible. For each list, the total number of correct responses (1 point for accurate reading) was scored and compared to the norms for each school grade.

#### 2.3.2. Word Spelling (Accuracy)

Spelling accuracy was assessed with a standardised dictation task from the BALE [62], which was composed of three lists of words: 10 irregular, 10 complex regular and 10 simple regular words. The dictation task was not time-constrained, and the experimenter ensured that each child was ready before proceeding to the next word. A small break of two minutes was proposed to the child between the lists. One point was given for accurate spelling, and the children’s raw scores were compared to the norms for their grade given by the test. The scores on the three lists were summed, leading to a maximum score of 30 for word spelling.

#### 2.3.3. Handwriting (Legibility and Speed)

Participants’ handwriting was assessed with a text copying task taken from a standardised test in a limited time of five minutes (BHK, (Concise Evaluation Scale for Children’s Handwriting); [63]). The quality of the handwriting was scored through the use of precise aesthetic criteria (e.g., the sizes of the letters, correction of letterforms, letter distortion). Each sentence was scored according to those criteria. The presence of one of the graphical errors led to assigning 1 point. The raw scores were then compared to test age norms. The number of letters correctly copied was scored and then compared to test age norms as an indicator of handwriting speed. The task had a very high reported interrater reliability (*r =* 0.90), and the internal reliability in the current sample given by Cronbach’s alpha was 0.83.

### 2.4. MRI Scanning

#### 2.4.1. Imaging Acquisition Parameters

All participants underwent an MRI session during which three-dimensional (3D) T1-weighted and resting-state functional MRI (RS-fMRI) T2*-weighted sequences were acquired. The data were collected using a 3T MRI (Signa^TM^ Premier, General Electric Company, Milwaukee, WI, USA) equipped with a 48-channel coil and installed at the Cliniques Universitaires Saint-Luc (UCLouvain, Brussels, Belgium). The 3D T1 encompassing the whole brain was selected to provide detailed anatomy (1 mm³) due to an MPRAGE sequence (inversion time = 900 ms; repetition time (TR) = 2188.16 ms; echo time (TE) = 2.96 ms; flip angle (FA) = 8°; field of view (FOV) = 256 × 256 mm²; matrix size = 256 × 256; 156 slices; slice thickness = 1 mm; no gap; and total scan time = 5 min 36 s). The RS-fMRI sequences were collected with hyperband (Factor 3) echo-planar imaging: FOV= 220 × 220 mm²; matrix size = 110 × 110; TE = 30 ms; FA = 90°; slice order ascending and interleaved; slice thickness = 2 mm; and ARC 2 (parallel imaging). The TR was 1500 ms and the number of slices was 64, with the whole brain scanned 250 times per run (=6 min 15 s).

#### 2.4.2. MRI Data (Pre)Processing

The MRI data were analysed using BrainVoyager (Version 20.4 for Windows, Brain Innovation, Maastricht, The Netherlands). Preprocessing of the resting-state data consisted of linear trend removal to exclude scanner-related signal drift, a temporal high-pass filter to remove frequencies lower than 0.005 Hz and correction for head movements using a rigid body algorithm for rotating and translating each functional volume in 3D space. The data were also corrected for time differences in the acquisition of the different slices. The data were co-registered with their 3D T1-weighted scans and normalised in MNI space. All co-registrations were verified, and movement corrections were optimised using sinc interpolation. As spontaneous low-frequency fluctuations are not exclusively BOLD-related fluctuations but are also contaminated by nonneural signals (i.e., artefacts), several additional preprocessing steps were added to remove these undesirable sources of variance. Regression analyses were performed to remove artefacts due to residual motion (the six movement regressors were obtained during the previous motion correction) and changes in ventricles (the signal from the ventricular mask defined in each participant). The final data were smoothed in the spatial domain (Gaussian filter: full width at half maximum = 5 mm).

RSFC was analysed using seed-voxel correlation mapping, with the GMFA as the seed region. This region was selected because of its key role in graphomotor processes. More precisely, the GMFA has been shown to be the interface between orthographic information and motor programmes that are specific to handwriting [26]. The seed region was created on BrainVoyager with a radius of 5 mm centred on the MNI coordinates reported in a meta-analysis by Planton and colleagues [27] (x = −22, y = −8, z = 54).

#### 2.4.3. Statistical Analyses

For each participant, we generated first-level connectivity maps between the seed region and all other voxels of the brain. Then, we entered these connectivity maps in a whole-brain ANOVA to investigate the effect of groups as a between factor (DYS; TD). This analysis allowed us to identify the brain regions with which the GMFA correlates differently depending on children’s group (DYS; TD). Last, we focused on understanding the relationships between RSFC and behavioural performance using standardised scores. As a reminder, TD children significantly outperformed DYS children in spelling, handwriting and reading measures (see Table 1). Correlational analyses using Pearson’s *r* coefficient were conducted between behavioural measures and RSFC between GMFA and the clusters for which significant differences between TD and DYS were revealed. These analyses were informative regarding the strength and direction of association between each behaviour and RSFC. Finally, whole-brain ANCOVAs were computed for each writing behaviour (simple-regular word spelling accuracy, handwriting legibility) and for regular-word reading accuracy. These ANCOVAs allowed us to identify whether the group differences in RSFC between TD and DYS children were still present when their performances in spelling, handwriting and reading were entered in the analysis. 

## 3. Results

### 3.1. Differences in Connectivity between DYS and TD Children (Whole-Brain ANOVAs with the GMFA Seed Region)

**Figure 1:**
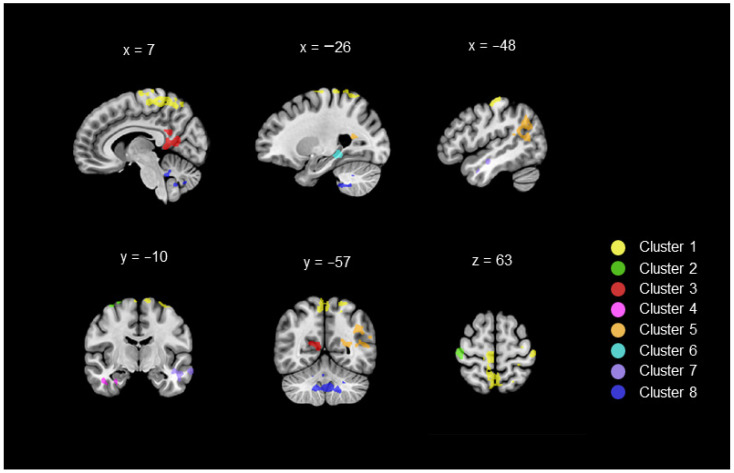
Visual representation of the eight clusters for which the GMFA seed region showed reduced RSFC in DYS compared to TD (whole-brain ANOVA, *p* < 0.05 corrected for multiple comparison).

**Figure A1:**
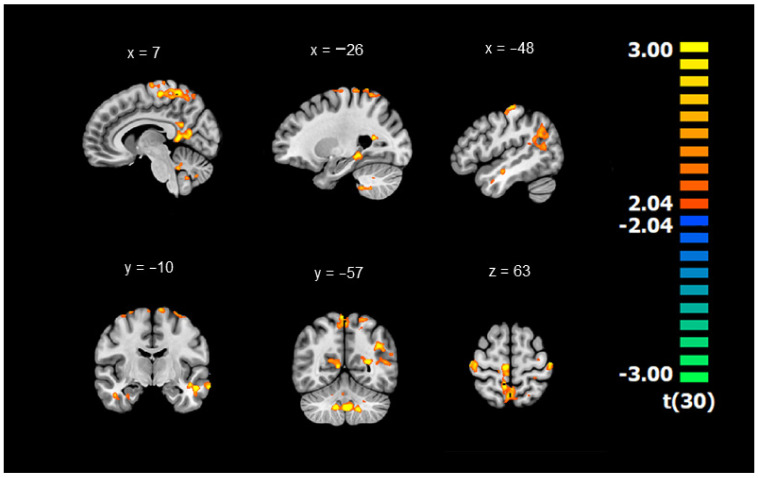
Visual representation of the clusters for which the GMFA seed region showed reduced RSFC in DYS compared to TD (whole-brain ANOVA, *p* < 0.05 corrected for multiple comparison).

Whole-brain ANOVAs revealed significant group differences for RSFC between the GMFA seed region and several brain regions. DYS and TD children exhibited different RSFCs between the GMFA and eight brain clusters (*p* < 0.05, corrected for multiple comparisons, cluster-level significance threshold = 206 voxels, see Figure A1 in Appendix A). A visual representation of these eight clusters can be found in Figure 1. Table 2 reports the MNI coordinates of the eight clusters, as well as the peaks contained inside of each, representing brain areas with which the GMFA connects differently at rest depending on children’s group. Notably, all the significant results reflected a reduction in RSFC in DYS children compared to TD children.

Group differences were found within the frontoparietal regions (Clusters 1 and 2), which comprehended regions of the paracentral lobules, regions of the postcentral and precentral gyri, regions of the precuneus, the left superior frontal gyrus and the right medial frontal gyrus. DYS children also showed reduced RSFC compared with TD children between the GMFA and limbic regions (Clusters 3 and 4), which comprehended the bilateral posterior cingulate region and parts of the parahippocampal gyrus, including the amygdala. The statistical analyses revealed that DYS children showed less RSFC between the GMFA and temporolimbic regions than TD children (Clusters 5 and 6), which comprehended part of the left superior temporal gyrus, left parahippocampal gyrus, including the hippocampus, and left fusiform gyrus. Group differences in RSFC between the GMFA and temporal regions (Cluster 7) were also revealed in regions of the left hemisphere: superior temporal, fusiform and middle temporal gyri. The last cluster for which DYS children exhibited significantly lower RSFC than TD children involved the cerebellar regions (Cluster 8). This cluster included bilateral regions of the posterior cerebellar lobe, including cerebellar lobules VIIIa, IX and X and right regions of the anterior cerebellar lobe, including Crus I and lobule V.

### 3.2. RSFC–Behaviour Relationships (Whole-Brain ANCOVAs with the GMFA Seed Region)

Behavioural scores in regular-word spelling accuracy (SP), handwriting legibility (HW) and regular-word reading accuracy (READ) (see Table 1) were used to analyse the RSFC–behaviour relationships. These scores were entered in Pearson bivariate correlational analyses for all children together and in each group separately (i.e., DYS and TD). Then, these scores were entered as covariates in the statistical analyses. These correlational and ANCOVA results are presented in Table 3 and Table 4, respectively. To keep this report short, we only presented the results in the eight clusters highlighted by the whole-brain ANOVAs without focusing on all the peaks reported in Table 2. 

The correlational results revealed significant associations between children’s behavioural performances and RSFC between the GMFA and the eight clusters. First, one can notice that the three behaviours (SP, HW, READ) were positively significantly correlated with each other. When taking all children together, several clusters were significantly associated with the three behaviours (Clusters 3, 4, 7, 8). In contrast, RSFC between GMFA and Clusters 2 and 6 was only significantly correlated with READ. When conducted in each group separately, the correlation coefficients between the behaviours and RSFC in the eight clusters did not reach significance. However, one can notice several high negative values in the DYS group involving SP performance and Clusters 1 and 2 (*r* = −0.480 and *r* = −0.388, respectively). In contrast, all correlational values in the TD group were strictly positive. In other words, while greater RSFC between GMFA and the frontoparietal regions seemed to be associated with better spelling skills in typical development, DYS children showed a different pattern of association. 

Whole-brain ANCOVAs revealed the brain regions with which the GMFA no longer showed reduced RSFC in DYS children compared to TD children when considering their performance in either SP, HW or both. Overall, our analyses demonstrated that children’s SP, HW and READ abilities played a role in the group differences highlighted by the whole-brain ANOVAs (Table 2). More precisely, while SP and READ seemed to contribute to all the significant differences in RSFC between DYS and TD children, entering HW scores did not cause the group differences in the frontoparietal regions (Clusters 1 and 2) and temporolimbic regions (Cluster 6) to disappear. When both SP and HW were entered together in the analyses, the group differences between DYS and TD children in the eight clusters were no longer significant.

## 4. Discussion

The present study aimed to investigate writing difficulties in children with dyslexia, with a particular focus on their graphomotor abilities. First and foremost, it is essential to specify that the DYS children who participated in the current study had a severe spelling deficit and less legible handwriting than TD children. In addition to the behavioural assessment, resting-state functional connectivity (RSFC) was measured in all participants. These RSFC analyses focused on the left GMFA seed region, which is located in the left premotor cortex (BA6) in the posterior part of the middle frontal gyrus. Due to its role in transforming orthographic code in graphic traces [26], the left GMFA is a region that specialises in graphomotor processes. Our main findings concerned altered functional coupling between the GMFA and brain areas that play a role in literacy-related or motor-related skills (i.e., temporal areas; frontoparietal areas and cerebellar areas, respectively) in DYS children. Moreover, spelling and handwriting behavioural scores were entered as covariates in the analyses. This step demonstrated that the differences in RSFC with the GMFA observed between DYS and TD children were no longer significant when children’s performances in writing were considered. These results suggested that connectivity with the GMFA is a marker of writing impairment. In line with our predictions, the statistical analyses revealed graphomotor dysfunction in dyslexia in addition to spelling impairment. Additionally, our analyses revealed reduced connectivity in DYS children between the GMFA and the limbic system, which is known for its role in emotional and behavioural regulation. Altogether, these findings highlighted a disruption in the functional organisation of the handwriting circuit for the first time, which is already present in young DYS children and is evident at rest.

### 4.1. Connectivity Differences with the GMFA in Regions Involved in Phonological and Lexical Processes (Temporal Areas)

In accordance with our predictions, our group comparison analyses revealed that the left GMFA had reduced connectivity with temporal brain areas in DYS children compared to TD children (i.e., BA20, 21, 38 and 39) for which abnormalities in dyslexia have already been reported in past literature. In a coherent way, the correlational analyses demonstrated that better spelling, handwriting and reading skills were associated with greater strength of RSFC between the GMFA and temporal areas. These regions are known for their involvement in both procedures of reading and spelling, namely the lexical and phonological routes [64].

Indeed, differences in RSFC between DYS and TD children were highlighted in the left fusiform gyrus (BA20). Abnormalities in the fusiform gyrus have been repeatedly discussed in past literature, with both structural and functional results [42,43,44,45], rendering it a reliable marker of dyslexia. In particular, dyslexia is characterised by the dysfunction of the visual-word form area (VWFA), which is responsible for the lexical route of reading. Indeed, the left VWFA enables readers to directly access and quickly retrieve word orthographic forms during literacy tasks [65]. Interestingly, VWFA activity is known to increase with age and reading expertise [66]. Our study reveals that differences in connectivity between a key reading region and a key graphomotor region (VWFA and GMFA, respectively) were already present in our young sample of children (*M_age_* = 9.3 years). Our results also highlighted a reduction in RSFC in regions of the left middle temporal gyrus in DYS children, which is in line with the findings of a previous resting-state fMRI study in which reduced connectivity between the middle temporal gyrus and the right frontal pole was demonstrated [23]. The function of the middle temporal gyrus is crucial for reading as it is responsible for the retrieval of visually presented items [21].

Additional group differences in RSFC were highlighted in the superior temporal gyrus (BA38 and 39), which is critically involved in the phonological procedure of reading. Structural abnormalities such as decreased grey matter volume and dysfunction in DYS samples have already been revealed in BA38 [67]. Similarly, hypoactivation in the angular gyrus (BA39) has been demonstrated in DYS children compared to TD children [68,69], especially during reading tasks involving phonological processing [70].

Overall, our results reveal that the left GMFA, a region that is specialised in graphomotor processes, has reduced connectivity with regions that are recruited during reading and spelling for both phonological and lexical processes in DYS children [64]. These differences are visible at rest in primary school children.

### 4.2. Connectivity Differences with the GMFA in Motor Regions (Frontoparietal and Cerebellar Areas)

Our statistical analyses highlighted that the left GMFA connected differently with motor-related areas of the frontoparietal brain. First, the correlational analyses suggested a reverse pattern of association between behavioural performances and RSFC with frontoparietal areas depending on children’s group. Indeed, while typical development seemed to be associated with positive relationships between RSFC and reading and writing skills, negative associations were found in the DYS group. However, these negative associations did not reach significance, which makes them hard to interpret. Second, the group comparison analyses highlighted a significant reduction in RSFC with regions of the primary somatosensory cortices (left BA2, right BA3), the primary motor cortex (bilateral BA4, M1), somatosensory association cortices (bilateral BA5, 7) and the premotor and supplementary motor cortices (left BA6, SMA). These results are in line with our prediction, suggesting that literacy impairment in DYS children is not sufficient for explaining handwriting difficulties.

In particular, our results highlighted reduced RSFC between the GMFA and motor regions of the handwriting network in DYS children compared to TD children [27]. More precisely, a significant difference concerned the left precentral gyrus (BA4), which corresponds to the primary motor area (M1) in the handwriting network [27]. We also found differences in RSFC with a region of the left paracentral lobule (BA6), which corresponds to the supplementary motor area. Due to its role in the planning and execution of voluntary hand movements, this area is also part of the handwriting network [27]. These areas are crucial for handwriting but are not specific to graphomotor production as they are recruited for various motor-related tasks. Interestingly, no difference in fMRI activity was found for these specific motor regions of the writing network (i.e., M1 and SMA) between DYS and TD children during an fMRI word-dictation task [19]. Therefore, while past findings have suggested that these motor-related regions were not recruited differently during writing by DYS children, our results suggest that differences exist between DYS and TD children in the functional coupling between motor regions at rest.

Another main cluster of differences in RSFC between DYS and TD children was found in the cerebellum. Indeed, our results revealed reduced connectivity between the GMFA and bilateral posterior cerebellar regions and right anterior cerebellar regions in DYS children. These findings are in line with our predictions, revealing altered functional coupling between graphomotor regions. Indeed, the role of the right cerebellum in the control of complex finger movements has been highlighted for handwriting [27]. The major role of the cerebellum in the acquisition of writing skills has been confirmed in a recent task-fMRI study conducted in both children and adults [55]. Moreover, dysfunction of anterior and posterior regions in the right hemisphere was highlighted in DYS children during a dictation task in a recent fMRI study [19]. More broadly, cerebellar function in dyslexia has been the topic of many investigations. Indeed, past works have repeatedly highlighted functional and structural abnormalities of the cerebellum in DYS samples [71,72,73,74]. Therefore, observing RSFC reduction with the GMFA in cerebellar regions was not surprising. Such findings reinforce the hypothesis of graphomotor impairment in DYS children. Moreover, the present study adds additional evidence of the presence of cerebellar abnormalities as reliable markers of dyslexia [74], which were already noticeable in children at rest.

All these findings highlighted RSFC disruption in DYS children in regions involved in motor processes, which are known to be crucial for handwriting. In line with our hypothesis, these results support the idea that reduced connectivity between the GMFA and motor-related regions is a sign of graphomotor impairment in DYS children.

### 4.3. Connectivity Differences with the GMFA in Regions Involved in Emotion and Behaviour Regulation (Limbic System)

Our results revealed that the left GMFA had reduced connectivity with brain areas of the limbic system in DYS children compared to TD children, which is responsible for emotion and behaviour regulation. Moreover, the correlational analyses demonstrated that better performances in reading and writing were associated with greater RSFC with limbic regions. These findings were not expected as our research question specifically addressed DYS children’s writing difficulties. However, they are in line with past findings highlighting emotional struggles such as low self-esteem and anxiety issues in individuals with dyslexia [75,76].

Specifically, our results concerned areas of the posterior cingulate gyrus (BA29-31), the parahippocampal gyrus (BA34) and the amygdala. Recently, authors have examined the emotional network, i.e., the limbic system, in DYS children [77]. More precisely, their findings highlighted decreased functional connectivity between the amygdala and bilateral frontal pole regions. Our results revealed a similar pattern of reduced RSFC between the amygdala and the GMFA area in DYS children. With regard to the posterior cingulate cortex, its functions are recognised for various cognitive processes, such as memory retrieval, planning and control of attentional focus [78]. Altered connectivity in the posterior cingulate cortex has already been demonstrated in DYS children [66]. In this previous study, typical readers exhibited stronger RSFC between regions of the posterior cingulate and the left fusiform gyrus, which plays a key role in the lexical processes of reading (see 5.1.). Our findings extend these results by pointing to reduced RSFC in the posterior cingulate in relation to graphomotor processes in DYS children.

To the best of our knowledge, the limbic system has never been studied in relation to writing abilities in neuroimaging experiments. We propose that the significant differences in connectivity between a region specialised in graphomotor processes (the GMFA) and limbic regions may be proof of differences in emotional experiences during handwriting tasks in DYS children compared to TD children. Indeed, one might plausibly believe that the negative experiences of DYS children while learning to write significantly affect how these regions communicate. Knowing that our study was exclusively focused on understanding writing difficulties, the behavioural assessment did not include emotional measures. Such measures would have been useful to strengthen our interpretation of how negative emotions may significantly induce disruption in functional connectivity between the GMFA and the limbic system.

### 4.4. Synthesis: Are There Functional Markers of Graphomotor Impairment at Rest in DYS Children?

Overall, the RSFC results presented in the sections above revealed functional markers of graphomotor impairment at rest in DYS children. Indeed, our findings provided evidence that the writing difficulties encountered by DYS participants were not limited to spelling processes. Importantly, we demonstrated that the GMFA, a key graphomotor brain area, exhibited altered RSFC with not only spelling regions but also motor regions. Overall, we demonstrated for the first time that the behavioural manifestations of dyslexia (i.e., poor spelling, reading and handwriting performances) were associated with reduced RSFC with the GMFA region. In particular, reduced RSFC was demonstrated with motor areas crucial for handwriting, namely the primary motor area, the supplementary motor area and the right cerebellum. Additionally, our results suggested that emotional experiences in DYS children during writing development significantly impacted the functional coupling of the writing network and the limbic system.

Regarding the origin of DYS children’s handwriting difficulties, these findings must be interpreted cautiously. While our results constitute robust evidence of altered RSFC between regions involved in graphomotor production, they cannot ascertain the origin of this graphomotor dysfunction. Indeed, our findings do not exclude the possibility that graphomotor dysfunction is a consequence of deviant literacy development [9,11,18]. Indeed, knowing that the GMFA is the interface between orthographic and graphomotor processes, the GMFA receives inputs from spelling regions. Moreover, past longitudinal research in preliterate children has revealed that brain markers of literacy disability are already present in crucial phonological regions before reading and writing instruction in children who later develop dyslexia [79]. Knowing that DYS children have pre-existing dysfunction in spelling regions, the altered connectivity between spelling regions and the GMFA may subsequently damage connections with motor regions.

Notably, the differences in RSFC between the GMFA and the motor regions located in the frontoparietal cortex between DYS and TD children were no longer significant when spelling performance was entered in the analysis. In contrast, the reduced RSFC observed in the DYS sample between the GMFA and these motor regions remained significant when we considered children’s handwriting scores. One could speculate that these different contributions of spelling and handwriting to RSFC in the motor regions highlighted that altered RSFC in DYS children may be linked to spelling impairment and not to handwriting difficulties.

Finally, considering our findings in relation to the concept of orthographic–motor integration is interesting [80]. Knowing that the function of the GMFA is to bridge orthographic and motor information, the GMFA may be the region responsible for the ability of orthographic–motor integration. Revealing reduced RSFC with the GMFA from various areas of the brain may be interpreted as a sign of a deficit in orthographic–motor integration in dyslexia, as suggested by authors [10].

More research is needed to understand whether graphomotor impairment in dyslexia is independent of literacy deficits or its consequences. Nevertheless, the current study demonstrated that young DYS children already show disorganisation of the handwriting network even at rest and that these abnormalities concern graphomotor regions and not just spelling regions. Overall, this study supports the hypothesis of the presence of graphomotor impairment in DYS children in addition to spelling deficits.

### 4.5. Study Limitations and Perspectives

As is often the case in experiments involving neuroimaging data, the sample size in the current study was quite limited (*n* = 32), although it was consistent with the standards of other fMRI studies in individuals with and without dyslexia (e.g., *n* = 29 [23]; *n* = 15 [24]). Therefore, conducting similar analyses in resting-state data acquired in larger samples of DYS children is required to reinforce our conclusions. Furthermore, recruiting larger samples of children could allow comparison between DYS children and several groups of TD children, matched on various abilities and not just on chronological age. In particular, it would be interesting to compare DYS to several groups of TD children, being equivalent in literacy abilities and in fine motor skills. Designing future studies that present such methodological assets would greatly contribute to understanding the origin of handwriting difficulties in dyslexia. Moreover, given that the participants in this study were primary school children, importantly, they were still developing their spelling and graphomotor abilities [38]. Thus, handwriting difficulties in DYS children may be greater at the beginning of learning to write because both spelling and graphomotor processes require substantially more effort at this age. Therefore, measuring RSFC between the GMFA and motor regions in adult samples may help understand whether graphomotor difficulties in dyslexia persist into adulthood or if they only pertain to the arduous beginning of writing development. 

Another limitation of the present study concerns the behavioural assessment, which was not sufficiently comprehensive. Indeed, our measures exclusively focused on reading, spelling and handwriting abilities. Inevitably, even if scoring of the handwriting task was focused only on graphomotor skills, this copying task inevitably involved spelling processes in addition to graphomotor processes. To refine our understanding of the origin of graphomotor difficulties in dyslexia, future research must assess participants’ performances in various motor tasks, such as manual dexterity or visuomotor integration [81,82]. Indeed, investigating motor abilities in DYS children during tasks that do not recruit spelling processes is necessary to understand whether graphomotor impairment could be independent of spelling impairment. In the same line, adding an evaluation of the emotional variables in relation to writing would have been interesting. Indeed, our results highlighted altered RSFC with the limbic system, which suggested the importance of emotional factors during writing development [13]. Knowing that significant influences exist between motivational factors and writing performances [83,84], we can postulate that a vicious cycle exists between writing abilities and emotional factors. In other words, repeated episodes of writing failure may lead to increased negative emotions in relation to writing, which may further aggravate children’s writing performance. Our results encourage future research to focus on the role played by emotional factors in writing disability.

Despite these limitations, these studies revealed that young DYS children in primary school already present markers of graphomotor dysfunction. These results convey educational and clinical implications for practitioners working with DYS patients. This study draws attention to the fact that during handwriting, DYS children may face an even greater challenge than the one induced by their severe spelling deficit. Indeed, the current study supports the idea of additional graphomotor impairment in dyslexia. Future studies should test whether graphomotor instruction has the potential to help DYS children develop writing skills. Such interventional designs may greatly contribute to improving the current therapy practices used with DYS children.

## 5. Conclusions

For the first time, the issue of graphomotor impairment in dyslexia was addressed with a resting-state fMRI study. The present study revealed altered functional connectivity in dyslexia between the region responsible for transforming orthographic code into graphic traces (i.e., the left GMFA) and both spelling and motor regions. Overall, the present findings demonstrated markers of graphomotor impairment in dyslexia that are visible at rest and are already present in young DYS children. While the spelling difficulties encountered by DYS children are well documented, this study reinforces the need to focus on the graphomotor side of writing during literacy development in children with dyslexia. Indeed, our data indicate that dysfunction in the graphomotor circuit is already present in DYS children in the early stages of learning to write.

## Figures and Tables

**Table 1:** Participants scores on behavioural tasks and statistical group comparison.

	DYS				TD				Group Comparison		
	(*n* = 16; 5 Girls, 9 Boys)				(*n* = 16; 9 Girls, 5 Boys)						
	*M*	*SD*	*Min*	*Max*	*M*	*SD*	*Min*	*Max*	*t*(30)	*p*	
Reading accuracy											
Non-words raw (/20)	12.60	3.52	7.00	18.00	16.94	2.05	12.00	20.00	−4.23	<0.001	DYS < TD
Non-words standardised	−1.38	1.44	−4.41	0.21	−0.15	0.71	−1.99	1.25	−3.04	0.005	DYS < TD
Regular words raw (/20)	17.60	2.75	9.00	20.00	19.38	1.26	16.00	20.00	−2.34	0.027	DYS < TD
Regular words standardised	−1.36	1.39	−3.56	0.45	0.01	0.89	−1.88	0.77	−3.27	<0.001	DYS < TD
Irregular words raw (/20)	14.93	4.04	3.00	19.00	18.00	2.61	12.00	20.00	−2.53	0.017	DYS < TD
Irregular words standardised	−1.24	1.17	−3.41	0.49	0.27	0.75	−1.03	1.14	−4.28	<0.001	DYS < TD
Spelling accuracy											
Simple regular words raw (/10)	6.81	2.66	0.00	10.00	8.94	1.18	7.00	10.00	−2.92	0.010	DYS < TD
Standardised	−2.04	2.03	−5.48	0.87	−0.11	0.87	−1.78	0.87	−3.49	<0.001	DYS < TD
Complex regular words raw (/10)	5.19	3.10	0.00	10.00	7.81	2.26	4.00	10.00	−2.74	0.010	DYS < TD
Standardised	−2.52	2.32	−8.46	0.95	−0.46	1.13	−1.93	0.95	−3.18	<0.001	DYS < TD
Irregular words raw (/10)	3.44	2.03	0.00	8.00	6.94	3.21	2.00	10.00	−3.68	0.002	DYS < TD
Standardised	−2.08	1.05	−4.44	−0.44	−0.08	1.11	−1.79	1.24	−5.22	0.003	DYS < TD
Handwriting legibility											
Raw (number of errors)^a^	15.31	7.81	39.00	8.00	9.55	4.26	2.00	17.00	2.59	0.015	DYS < TD
Standardised	−0.68	1.57	−4.44	0.89	0.82	1.31	−1.29	3.57	−2.93	0.006	DYS < TD
Handwriting speed											
Raw (number of words copied)	186.75	63.91	82.00	273.00	223.38	83.05	102.00	393.00	−1.40	0.172	DYS = TD
Standardised	−0.30	0.73	−1.48	0.62	0.31	1.28	−1.85	3.18	−1.65	0.110	DYS = TD

Note. Standardised scores correspond to participants’ scores compared to test age or grade norms. The standardised scores are z-scores (*M* = 0; *SD* = 1). Scores below −2 *SD* were considered. DYS = children with dyslexia, TD = typically-developing children, *M* = mean, *SD* = standard deviation.

**Table 2:** Significant effect of group for the connectivity maps of the GMFA seed region (whole-brain ANOVAs).

Correlated Brain Area (DYS < TD)					MNI Peak			
Size					x	y	z	*t*(30)
13,639	Cluster 1	Bilateral Frontoparietal			−11	−36	68	2.43
		L	Paracentral Lobule	BA 6	−4	−25	69	3.04
		L	Postcentral Gyrus	BA 2	−49	−22	63	2.88
		L	Postcentral Gyrus	BA 5	−18	−35	76	3.04
		L	Postcentral Gyrus	BA 5	−33	−35	71	3.09
		L	Precentral Gyrus	BA 4	−36	−22	69	2.81
		L	Precentral Gyrus	BA 4	−37	−16	67	2.80
		L	Precuneus	BA 7	2	−57	67	2.99
		L	Superior Frontal Gyrus	BA 6	−21	−3	76	2.82
		R	Medial Frontal Gyrus	BA 6	5	−13	74	2.80
		R	Paracentral Lobule	BA 5	8	−39	59	2.83
		R	Paracentral Lobule	BA 6	6	−24	64	3.05
		R	Postcentral Gyrus	BA 7	25	−49	74	3.11
		R	Precuneus	BA 7	8	−47	65	3.07
2943	Cluster 2	Right Frontoparietal			40	−21	65	2.38
		R	Postcentral Gyrus	BA 3	43	−25	65	2.85
		R	Postcentral Gyrus	BA 3	34	−26	72	2.88
		R	Precentral Gyrus	BA 4	32	−16	72	2.84
4263	Cluster 3	Bilateral Limbic			6	−52	13	2.49
		L	Posterior Cingulate	BA 29	0	−46	7	3.09
		R	Posterior Cingulate	BA 30	19	−62	14	2.95
		R	Posterior Cingulate	BA 30	7	−60	12	2.90
		R	Posterior Cingulate	BA 30	6	−51	24	2.85
1693	Cluster 4	Right Limbic		BA 34	37	−3	−28	2.32
		R	Parahippocampal Gyrus	BA 34	33	1	−28	2.85
		R	Parahippocampal Gyrus	Amygdala	33	−4	−22	2.91
5940	Cluster 5	Left Temporolimbic			−39	−56	23	2.39
		L	Posterior Cingulate	BA 31	−15	−50	23	3.05
		L	Superior Temporal Gyrus	BA 39	−43	−56	33	2.91
1766	Cluster 6	Left Temporolimbic			−31	−35	−15	2.44
		L	Parahippocampal Gyrus	Hippocampus	−28	−37	−8	3.12
		L	Parahippocampal Gyrus	BA 35	−32	−31	−24	2.86
		L	Fusiform Gyrus	BA 20	−35	−38	−17	2.83
3621	Cluster 7	Left Temporal			−58	−9	−18	2.48
		L	Superior Temporal Gyrus	BA 38	−40	20	−36	2.92
		L	Fusiform Gyrus	BA 20	−40	−5	−27	3.34
		L	Sub-Gyral	BA 21	−46	−11	−16	2.91
		L	Middle Temporal Gyrus	BA 21	−55	−4	−18	3.10
		L	Middle Temporal Gyrus	BA 21	−65	−3	−18	2.82
		L	Middle Temporal Gyrus	BA 21	−68	−13	−16	3.22
7273	Cluster 8	Bilateral Cerebellum			4	−52	−41	2.52
		L	Posterior Lobe	Lob X	−22	−41	−46	2.43
		L	Posterior Lobe	Lob IX	−6	−55	−42	2.73
		R	Posterior Lobe	Lob IX	10	−54	−42	2.52
		R	Posterior Lobe	Dentate	17	−52	−34	2.29
		R	Anterior lobe	Lob V	17	−51	−27	2.20
		R	Posterior Lobe	Lob X	24	−39	−44	2.51
		R	Posterior Lobe	Lob VIIIa	32	−45	−47	2.50
		R	Anterior Lobe	Crus I	44	−42	−37	2.46

Note. L = left; R = right; BA = Brodmann area; Lob = lobule. Cluster size is expressed in mm^3^. DYS = children with dyslexia, TD = typically-developing children, MNI = Montreal Neurological Institute coordinate system.

**Table 3:** Pearson correlations between behavioural measures and RSFC between the GMFA seed region and the eight clusters.

	All Children (*n* = 32)			DYS (*n* = 16)			TD (*n* = 16)		
Measures	SP	HW	READ	SP	HW	READ	SP	HW	READ
SP	-			-			-		
HW	0.514 **	-		0.297	-		0.557 *	-	
READ	0.592 **	0.711 **	-	0.411	0.564 *	-	0.528 *	0.659 **	-
Cluster 1	0.168	0.302	0.346	−0.480	0.062	−0.125	0.105	0.051	0.210
Cluster 2	0.203	0.274	0.357 *	−0.388	−0.072	0.067	0.159	0.143	0.190
Cluster 3	0.353 *	0.378 *	0.422 *	0.104	0.269	0.217	0.157	0.029	0.146
Cluster 4	0.359 *	0.421 *	0.390 *	0.241	0.387	0.206	0.083	0.008	0.045
Cluster 5	0.345	0.406 *	0.428 *	0.006	0.322	0.305	0.229	0.138	0.148
Cluster 6	0.256	0.296	0.387 *	−0.099	−0.036	0.081	0.106	0.102	0.158
Cluster 7	0.360 *	0.353 *	0.388 *	−0.146	−0.067	0.006	0.313	0.199	0.081
Cluster 8	0.496 **	0.467 **	0.517 **	0.456	0.348	0.403	0.209	0.119	0.152

Note. SP = spelling score; HW = handwriting score; READ = reading score; Cluster 1 = bilateral frontoparietal; Cluster 2 = right frontoparietal; Cluster 3 = bilateral limbic; Cluster 4 = right limbic; Cluster 5 = left temporo-limbic; Cluster 6 = left temporo-limbic; Cluster 7 = left temporal; Cluster 8 = bilateral cerebellum. * *p* < 0.05; ** *p* < 0.01.

**Table 4:** Effect of group using spelling, handwriting and reading behaviours as covariates (whole-brain ANCOVAs with the GMFA seed region).

Correlated Brain Area (DYS < TD)		Cov SP	Cov HW	Cov SP and HW	Cov READ
		*t*(29)	*t*(29)	*t*(28)	*t*(29)
Cluster 1	Bilateral frontoparietal	2.00	2.33 *	2.04	1.86
Cluster 2	Right frontoparietal	1.92	2.13 *	1.87	1.62
Cluster 3	Bilateral limbic	1.86	1.95	1.67	1.67
Cluster 4	Right limbic	1.57	1.79	1.42	1.62
Cluster 5	Left temporolimbic	1.65	1.84	1.49	1.51
Cluster 6	Left temporolimbic	2.02	2.11 *	1.92	1.73
Cluster 7	Left temporal	2.03	2.03	1.85	1.92
Cluster 8	Bilateral cerebellum	1.75	1.76	1.47	1.49

Note. Cov = covariate; SP = spelling accuracy; HW = handwriting legibility; READ = reading accuracy. * *p* < 0.05.

## Data Availability

Ethical restrictions prevent sharing of any study data under any circumstances with anyone outside the author team (Ethics Committee of the University Hospital of Saint-Luc, Brussels, Belgium. Number: B403201942022).
